# Editorial: Baclofen in the Treatment of Alcohol Use Disorder

**DOI:** 10.3389/fpsyt.2019.00338

**Published:** 2019-05-15

**Authors:** Renaud de Beaurepaire, Mathis Heydtmann, Roberta Agabio

**Affiliations:** ^1^Groupe Hospitalier Paul-Guiraud, Villejuif, France; ^2^Department of Gastroenterology, Royal Alexandra Hospital Paisley, Paisley, United Kingdom; ^3^Section of Neuroscience and Clinical Pharmacology, Department of Biomedical Sciences, University of Cagliari, Cagliari, Italy

**Keywords:** alcohol use disorder, baclofen, prescription, safety, mode of action

Alcohol use disorder (AUD) is a severe illness for which available treatments are of limited efficacy. Over the last 20 years, baclofen has progressively emerged as a potentially useful treatment for AUD, but our knowledge on the best way to prescribe it, on potential influencing factors on its effects, and on its mechanism of action in AUD is still limited. Knowledge on its efficacy is also debated. As all three of us—RB, MH, and RA—have a long practice of baclofen use in the treatment of AUD, we thought of writing a special issue on this topic, and were delighted when *Frontiers in Psychiatry* accepted to give us this opportunity [two of us, RB and MH, have previously participated in the writing of a book on the same topic (1, 2)]. The realization of this new special issue has proved to be a kind of adventure we did not expect at the start.

We aimed at joining our competencies to provide a shared description of the methods to obtain an optimal therapeutic effect of baclofen in the treatment of AUD. We were aware that to obtain its therapeutic effect, the required dose of baclofen may largely vary among patients, certain patients requiring low doses and other high or very high doses. In other words, our experience showed that patients require “personalized doses” of baclofen that allow them to say “well, at this dose I have no more craving for alcohol, I do not experience craving when I see bottles or people who drink,” meaning that, following Olivier Ameisen’s words, the patient has reached a state of “indifference towards alcohol” (3).

Our special issue had three goals. The first was to give a general view of baclofen use for AUD treatment in different countries. The second was to provide joined information on the methods to prescribe baclofen in the treatment of AUD. The third was to obtain recent data from different research teams involved in various aspects of baclofen use in the treatment of AUD. To achieve these goals, we solicited the participation of a large number of baclofen prescribers worldwide.

For our first goal, Garbutt describes the use of baclofen in the US. According to this contribution, there is a limited use of baclofen to treat AUD in the US. However, Garbutt points out that there is, in general, a very low rate of medication use for the treatment of AUD in the US, largely due to a lack of knowledge of physicians and patients about the potential value of medications. Regarding baclofen, the results of meta-analyses showing that its efficacy is equivocal, the concerns of tolerability, and the fact that its prescription remains off label are all elements that may deter clinicians and patients. Garbutt nevertheless mentions that there are no accurate data on the use of baclofen in AUD in the US, making it difficult to know if clinicians prescribe it. These remarks probably apply to most other countries, except France, which is the only country where baclofen has an official approval from Health Authorities in the treatment of AUD, and where baclofen is more widely prescribed.

For our second goal, we convened a large group of international experts, representative of researchers and physicians who contributed to the recent studies on baclofen and AUD. This group of experts achieved a consensus on the use of baclofen to treat AUD—“the Cagliari Statement”—published by *Lancet Psychiatry* in 2018 (4). After this concise and rigorous document, the group decided to provide a more detailed description of the different methods used to administer baclofen in the treatment of AUD (de Beaurepaire et al.). In detail, we describe how, especially in experimental studies, baclofen is usually administered in fixed doses to evaluate the efficacy and safety of each specific dose whereas, in clinical practice, baclofen is usually administered in flexible doses. In other words, the dose is gradually increased until the patient achieves the desired effects or side effects that prevent a further increase in dose. In this article, other than this description of the methods adopted for baclofen administration, there is also an unprecedented exhaustive review of published studies, approved by 26 authors coming from seven different countries (Australia, France, Germany, Great Britain, Italy, The Netherlands, and the US).

For our third goal, several innovative reports dealing with baclofen prescription and use, pharmacokinetics, preclinical research, and clinical investigations aiming to understand its potential mechanisms of action in AUD are published in the special issue. Reports on baclofen prescription and use include a long-term retrospective study (Pinot et al.); a research report on the response to baclofen in patients receiving antidepressants (Heng et al.); reviews on the effects of baclofen in alcohol withdrawal syndrome (AWS) (Cooney et al.), on the adverse effects of baclofen (Rolland et al.), and on the management of self-poisoning with baclofen (Franchitto et al.); and reviews focused on comorbidities—with liver cirrhosis (Mosoni et al.) and other mental disorders (Agabio and Leggio). In a 3-year retrospective study, Pinot et al. have followed 144 patients with AUD receiving tailored doses of baclofen (50 to 520 mg/day; average dose, 211 mg/day), and the treatment was successful in 63.3% of the patients (according to the WHO classification criteria) (Pinot et al.), a percentage of success grossly similar to that reported in other previously published observational studies. Depression and antidepressant treatment are common features in patients with AUD, and it was important to search for a potential interaction between baclofen and antidepressants. Heng et al. show that in patients on long-term treatment with antidepressants prior to starting baclofen, a beneficial effect on drinking outcomes can be shown like in patients who are not using antidepressants. However, there was a trend to an interaction between the two that needs further investigations (baseline tobacco use or alcohol liver disease may interfere with the interaction) (Heng et al.). The effectiveness of baclofen in the treatment of AWS is controversial, and Cooney et al., in their review, confirm that the evidence does not support the use of baclofen as a first-line treatment of AWS. Adverse effects too often limit or circumvent baclofen treatment, and this issue must be seriously considered. Rolland et al. propose a thoughtful review on baclofen adverse effects, separating the common and benign ones (sedation, insomnia, dizziness, and tinnitus) from the rare but potentially dangerous ones (seizures, mania, and sleep apnea), and point that concurrent consumption of alcohol, benzodiazepines, and other sedatives may worsen many adverse effects. These considerations have consequences on baclofen treatment management in terms of prevention of adverse effects and obtaining an optimal response (Rolland et al.). Cases of intentional or non-intentional baclofen self-poisoning have increased in parallel with the increasing use of baclofen in AUD. These cases present with particular clinical features that clinicians must be aware of. Franchitto et al. review the literature on that subject and address the management of this condition. AUD is often associated with liver cirrhosis and/or mental illnesses. AUD is difficult to treat in patients with liver cirrhosis because approved AUD medications may impair liver function. Baclofen is attractive because it has no liver toxicity, and Mosoni et al. review the clinical trials investigating the effect of baclofen in patients with AUD associated with liver disease. The results show a very favorable effect of baclofen in these patients, although the most appropriate dose of the drug remains to be determined (Mosoni et al.). Mental illness may alter the outcome in baclofen-treated patients. Agabio and Leggio address this issue by using a narrative analysis of all clinical and human laboratory studies of baclofen treatment in patients with AUD with or without mental illness. The most frequent psychiatric comorbidities are anxiety and depression. Further work is needed to determine if these comorbidities interfere with baclofen treatment outcome (Agabio and Leggio).

Reports on baclofen pharmacokinetics include two studies by Simon et al. In their first article, Simon et al. investigate the pharmacokinetics of baclofen in 60 patients with AUD treated with various doses of baclofen (up to 300 mg/day). The results show that baclofen has a linear pharmacokinetic profile, corresponding to a one-compartment model, with no influencing clinical or biological factor (Simon et al.). In their second article, Simon et al. review the literature investigating the baclofen dose in relation to the pharmacokinetics of the drug. Indeed, the dose–response variability in baclofen-treated patients may be related to variability in pharmacokinetics. In particular, the effects of intestinal absorption, blood–brain barrier transport, and renal elimination are highlighted (Simon et al.).

Preclinical research is reviewed by Colombo and Gessa. Baclofen suppresses many alcohol-related behaviors in laboratory animals, including locomotor activity, alcohol drinking and self-administration, binge-like drinking, relapse drinking, alcohol seeking, and AWS symptoms. These effects of baclofen in animals provide interesting elements for the understanding of its mechanism of action in AUD (Colombo and Gessa). Two clinical studies also bring new data that could help the understanding of the mechanism of action of baclofen. Morley et al., using magnetic resonance spectroscopy, show that baclofen increases the concentrations of the antioxidants glutathione and *N*-acetyl aspartate in the brain of patients with AUD, and that higher glutathione levels predict favorable outcomes at follow-up. This supports the idea that the beneficial effects of baclofen in the treatment of AUD could be, at least in part, related to neuroprotective mechanisms (Morley et al.). Durant et al. show that the effects of an acute administration of baclofen are very different in patients with AUD compared with healthy controls. The measured effects were growth hormone release and subjective experience (for instance, feeling “drunk”, “dizzy”, or “stimulated”). All these responses were blunted in patients with AUD while they were very marked in healthy controls. According to the authors, these results indicate a lower sensitivity to baclofen and, by extension, a general lower GABA-B receptor sensitivity in patients with AUD (Durant et al.). Finally, the potential mechanisms of baclofen in AUD are reviewed in an article that concludes that baclofen may produce an indifference to alcohol by suppressing the Pavlovian association between alcohol cues and rewards through an action in a critical part of the dopaminergic network (the amygdala). This action of baclofen is made possible by the fact that baclofen and alcohol act on similar brain systems (in particular GABA-B systems) (de Beaurepaire).

We really hope that this special issue will contribute to improve our knowledge and enhance debates and research on the use of baclofen in the treatment of AUD, a devastating illness that is dramatically undertreated.

## Author Contributions

All authors equally contributed to this work.

## Conflict of Interest Statement

The authors declare that the research was conducted in the absence of any commercial or financial relationships that could be construed as a potential conflict of interest.

The reviewer GA declared a past co-authorship with one of the authors RA to the handling Editor.
